# RNA-Dependent RNA Polymerase from Heterobasidion RNA Virus 6 Is an Active Replicase In Vitro

**DOI:** 10.3390/v13091738

**Published:** 2021-08-31

**Authors:** Alesia A. Levanova, Eeva J. Vainio, Jarkko Hantula, Minna M. Poranen

**Affiliations:** 1 Molecular and Integrative Biosciences Research Programme, Faculty of Biological and Environmental Sciences, University of Helsinki, 00790 Helsinki, Finland; 2 Natural Resources Institute Finland, 00790 Helsinki, Finland; eeva.vainio@luke.fi (E.J.V.); jarkko.hantula@luke.fi (J.H.)

**Keywords:** viral RdRp, mycovirus, dsRNA virus, TNTase activity, replicase activity, semi-conservative transcription

## Abstract

Heterobasidion RNA virus 6 (HetRV6) is a double-stranded (ds)RNA mycovirus and a member of the recently established genus *Orthocurvulavirus* within the family *Orthocurvulaviridae*. The purpose of the study was to determine the biochemical requirements for RNA synthesis catalyzed by HetRV6 RNA-dependent RNA polymerase (RdRp). HetRV6 RdRp was expressed in *Escherichia coli* and isolated to near homogeneity using liquid chromatography. The enzyme activities were studied in vitro using radiolabeled UTP. The HetRV6 RdRp was able to initiate RNA synthesis in a primer-independent manner using both virus-related and heterologous single-stranded (ss)RNA templates, with a polymerization rate of about 46 nt/min under optimal NTP concentration and temperature. NTPs with 2′-fluoro modifications were also accepted as substrates in the HetRV6 RdRp-catalyzed RNA polymerization reaction. HetRV6 RdRp transcribed viral RNA genome via semi-conservative mechanism. Furthermore, the enzyme demonstrated terminal nucleotidyl transferase (TNTase) activity. Presence of Mn^2+^ was required for the HetRV6 RdRp catalyzed enzymatic activities. In summary, our study shows that HetRV6 RdRp is an active replicase in vitro that can be potentially used in biotechnological applications, molecular biology, and biomedicine.

## 1. Introduction

RNA-dependent RNA polymerases (RdRp) catalyze RNA synthesis using an RNA template, and they are indispensable for the life cycle of viruses with single-stranded (ss) and double-stranded (ds)RNA genomes. Apart from several short, conserved sequence motifs (motifs A–G [1,2,3]), RdRp from distantly related viruses do not share significant amino acid similarity. However, all structurally characterized viral RdRps have a similar cupped right-hand shape with palm, fingers, and thumb subdomains comprising a common structural core of 231 equivalent residues, which indicate their shared evolutionary origin [4,5].

All viral RdRps employ the universal two-metal-ion mechanism of nucleotide addition [6], in which two catalytically active divalent metal ions, coordinated by conserved aspartates in motifs A and C, are required for the RNA polymerization reaction. Additionally, a third divalent cation binding site has been observed in the high-resolution structures of several RdRps [7,8], including representatives from different families of positive-sense ssRNA [(+)RNA] and dsRNA viruses infecting both eukaryotic and prokaryotic hosts. This third ion has been shown to have an important role in the correct coordination of NTPs for catalysis [9].

Our current understanding of structures and biochemical properties of viral RdRps is heavily biased towards medically significant viruses and few bacterial RNA viruses. The importance of RdRps as targets for antiviral drugs development has been a key motivation to study viral RdRps. For instance, nucleoside analogues ribavirin, remdesivir, sofosbuvir, and favipiravir inhibit replication of a number of RNA viruses, including SARS-CoV-2 [10,11,12,13]. Basic knowledge on viral RdRps has promoted innovation in the field of RNA interference (RNAi) [14,15,16] and generation of attenuated vaccine strains having increased polymerization fidelity [17]. In addition, studies on RdRps have provided fundamental information on key biological concepts, such as nucleic acid synthesis and evolutionary origin of viruses. Currently there is structural information available on viral RdRps from 42 viral species representing 16 viral families (Protein Data Bank; https://www.wwpdb.org/ accessed on 30 June 2021) [4]. Apart from a few well-studied RdRps (e.g., RdRp from bacteriophages phi6 [18,19], phi8, phi13 [20], human picobirnavirus (PBV) [21], flaviviruses [22,23], and picornaviruses [24]), the in vitro activity of RdRps from some economically important plant viruses has been demonstrated [25,26,27], but the information on RdRps from fungal viruses is scarce. Currently, there are no high-resolution structures available for mycoviral polymerases and the biochemical data on transcription and replication activities of these enzymes are obtained from the studies on purified virions [28,29,30] or mitochondria fractions of infected cells [31].

To revise the existing viral RdRp diversity and to fill in gaps in knowledge on RdRps of fungal RNA viruses, we established expression systems for RdRps of two fungal dsRNA viruses, Heterobasidion RNA virus 6 (HetRV6) and Heterobasidion partitivirus 12 (HetPV12), and present biochemical characterization of the HetRV6 RdRp. These viruses represent different viral families, but both share a bi-segmented dsRNA genome and relatively simple capsid architecture. HetRV6 is responsible for the majority of dsRNA virus infections detected in *Heterobasidion* isolates [32], but also infections caused by partitiviruses and mitoviruses are frequently encountered [33,34,35]. Fungi from the *Heterobasidion* species complex are widely distributed in the coniferous forests of the Northern Hemisphere, and some *Heterobasidion* spp. are notorious pathogens of conifers causing a significant loss to forest owners [36]. HetRV6 was recently assigned to a new taxonomic family *Curvulaviridae*, genus *Orthocurvulavirus*. The genomes of orthocurvulaviruses comprise two dsRNA segments designated as dsRNA1 and dsRNA2 [37]. Based on the presence of conserved sequence motifs A to F [32], an open reading frame (ORF1) identified in HetRV6 dsRNA1 has been predicted to encode a putative RdRp of 606 amino acids. Phylogenetic analysis based on RdRp amino acid sequences suggest that curvulaviruses are related to the members of the family *Amalgaviridae* and of unassigned group of RNA viruses, “unirnaviruses” [37].

Here, we demonstrate that the isolated HetRV6 RdRp subunit is enzymatically active in vitro and describe biochemical requirements for the RNA-directed RNA polymerization reaction. We show that HetRV6 RdRp possesses replicase and terminal nucleotidyl transferase (TNTase) activities, which are strongly dependent on the presence of Mn^2+^ ions. Our results suggest semi-conservative mode of transcription for HetRV6 polymerase. The HetRV6 RdRp is active on both homologous and heterologous templates. In addition to canonical NTPs, it can use 2′-fluoro-modified dNTPs for RNA polymerization. The data obtained provide the basis for in vitro application of HetRV6 RdRp to synthesize dsRNA molecules for biomedical and biotechnological purposes, which can be applied, for instance, to induce RNA interference (RNAi). To our knowledge, this is the first study on biochemical characterization of an isolated fungal virus RdRp.

## 2. Materials and Methods

### 2.1. Bacterial Strains and Plasmids

*Escherichia coli* DH5α (Gibco-BRL, Crewe, UK) served as the host for plasmid propagation and molecular cloning. *E. coli* BL21(DE3)pLysS strain ([38]; Novagen, Madison, WI, USA) was used for expression of viral RdRps. Plasmid pCR2.1-TOPO (Thermo Fisher Scientific, Carlsbad, CA, USA) containing a complementary DNA (cDNA) copy of the full-length dsRNA1 genome segment of curvulavirus strain HetRV6-ab6 (GenBank number HQ189459.1) [32] or alphapartitivirus strain HetPV12-an1 (GenBank number KF963175) was used to subclone RdRp gene into *E. coli* expression vector pET-32b(+) or pET-28a(+) (Novagen, Madison, WI, USA). Plasmid pCR2.1-TOPO_HetRV6_RNA1 [32] was also used for the production of HetRV6-specific (+)RNA molecules. Plasmids pLM659, pEM15, and pEM19 were applied to produce phi6-specific (+)RNA molecules, pMA-RQ_PBV2 to prepare a (+)RNA corresponding to the genomic dsRNA2 from human PBV, and pT7luc was used to generate non-viral sequences corresponding to the mRNA of firefly luciferase (Table 1).

### 2.2. Cloning, Expression and Purification of Full-Length RdRps

To construct expression plasmids, the predicted RdRp sequences from HetRV6 and HetPV12 viruses were amplified from pCR2.1-TOPO vectors (please see Section 2.1 for details) using high-fidelity Phusion DNA polymerase (Thermo Fischer Scientific, Carlsbad, CA, USA). For the primer sequences please refer to the Table S1. The PCR products were cut with FastDigest restriction enzymes *NcoI* and *HindIII* (Thermo Fischer Scientific, Carlsbad, CA, USA), purified with NucleoSpin Gel and PCR Clean-up kit (Macherey Nagel, Düren, Germany), and ligated with the large fragment of *NcoI-HindIII* digested and gel-purified expression vector pET-32b(+) (Novagen, Madison, WI, USA) to express proteins with His-tag at the N-terminus. Alternatively, RdRp gene from HetRV6 was inserted into pET-28a(+) vector (Novagen, Madison, WI, USA) to express RdRp without any fusion tags. The correct insert was verified with Sanger sequencing at DNA sequencing service of the University of Helsinki. Competent *E. coli* BL21(DE3)pLysS cells were transformed with the resultant plasmid pET-28a_HetRV6pol, pET-32b_HetRV6pol, or pET32b-HetPV12pol.

For RdRp expression, the cells harboring an expression plasmid were induced with 0.5 mM isopropyl-β-D-thiogalactopyranoside (IPTG, Thermo Fisher Scientific, Carlsbad, CA, USA) and incubated at 17 °C for 18 h. All the subsequent steps were performed at 4 °C. Bacteria were collected by centrifugation and resuspended in 1/200 culture volume of lysis buffer (50 mM sodium phosphate pH 8.0, 100 mM NaCl, 1 mM Pefablock). The cell suspension was sonicated on ice (ultrasonic processor UP400S, Dr. Hielscher GmbH, Teltow, Germany; three times 20 s, 70% amplitude/0.5 cycle with 15 s breaks between cycles), and the lysate was clarified by 1h ultracentrifugation at 40,000 g (rotor T-1270, Thermo Fisher Scientific, Carlsbad, CA, USA) with a subsequent filtration through a 0.45 µm sterile filter unit (Sartorius Stedim biotech, Aubagne, France). A HisTrap HP 1 mL column (GE Healthcare, Chicago, IL, USA) was applied to purify RdRps with His-tag using buffer A1 (50 mM sodium phosphate pH 8.0, 45 mM imidazole, 300 mM NaCl) and buffer B1 (50 mM sodium phosphate pH 8.0, 300 mM imidazole, 300 mM NaCl). A linear gradient from 0 to 100% buffer B at 1 mL/min was applied for protein elution.

Alternatively, to purify HetRV6 RdRp without His-tag, the clarified lysate was loaded onto a 1 mL HiTrap Heparin HP column (GE Healthcare, Chicago, IL, USA) and the unbound proteins were washed out with buffer A2 (50 mM sodium phosphate pH 8.0, 100 mM NaCl) until baseline stabilized. The bound proteins were eluted with a 35-mL linear gradient from 0% to 100% buffer B2 (50 mM sodium phosphate pH 8.0, 1 M NaCl) at 1 mL/min flow-rate. Fractions of 1 mL were collected and analyzed by sodium dodecyl sulfate-polyacrylamide gel electrophoresis (SDS-PAGE). Those containing HetRV6 RdRp were combined, diluted 10-fold with mQ water, and loaded onto 1 mL HiTrap Q HP anion exchange chromatography column (GE Healthcare, Chicago, IL, USA). A linear gradient of 35 mL from 0 to 100% buffer B2 was applied at 1 mL/min. For the final purification step, the fractions containing HetRV6 RdRp were combined and loaded onto HiLoad 26/600 Superdex 200 prep grade column (Cytiva, Marlborough, MA, USA). The proteins were eluted with buffer A2 at 1.5 mL/min. The RdRp-containing fractions were combined and concentrated with Amicon Ultra-15 centrifugal filter unit with nominal molecular weight limit of 30 kDa (Merck, Kenilworth, NJ, USA). The protein concentration was measured using Bradford assay [42].

The purified proteins were diluted to 0.3 mg/mL with a storage buffer (62.5% glycerol, 50 mM Hepes-KOH pH 8.0, 0.1 mM EDTA pH 8.0, 0.125% Triton X-100, 100mM NaCl, 2 mM MnCl_2_) and stored at −20 °C until use. Homogenity and identity of the purified polymerase was confirmed by SDS-PAGE in 16% acrylamide gel followed by Western Blot with rabbit polyclonal antibodies raised against HetRV6 RdRp (GeneCust, Boynes, France).

To evaluate whether purified RdRp from HetRV6 forms oligomers, we used globular proteins of the similar size, bovine serum albumin (Sigma-Aldrich, St. Louis, MO, USA) and in-house produced RdRp from phi6 [18]. The proteins were analyzed using size exclusion chromatography on Superdex 200 10/300GL column (GE Healthcare, Chicago, IL, USA) with isocratically applied buffer A3 (50 mM phosphate buffer pH 8.0, 150 mM NaCl) at 0.3 mL/min.

### 2.3. Production of Template RNA Molecules and Prediction of Their Secondary Structure

The template ssRNA molecules were produced in vitro by run-off transcription using T7 polymerase. To this end, target sequences for ssRNA production were PCR amplified from the plasmids described in the Section 2.1 (Table 1; for primer sequences refer to Table S1). The PCR products were purified with NucleoSpin Gel and PCR Clean-up kit (Macherey Nagel, Düren, Germany). Alternatively, plasmids pEM15 and pEM19 were digested with *SmaI* (Fermentas, Vilnius, Lithuania) to generate templates for the production of phi6(+)RNA ∆s^+^_13_ and ∆s^+^_HP_, respectively. The digested plasmids were purified with QIAquick PCR purification kit (Qiagen, Germantown, MD, USA). All produced dsDNA templates contained T7 polymerase promoter sequence at the 5′-end. SsRNAs were produced in 200 µL reaction mixtures containing 2 µg of template dsDNA, a mixture of four nucleotide triphosphates (NTPs; 5 mM each), 160 U of Ribolock (Thermo Fisher Scientific, Carlsbad, CA, USA), 800 U of T7 polymerase (Finnzymes, Helsinki, Finland), 120 mM Hepes-KOH pH 7.5, 24 mM MgCl_2_, 20 mM DTT, 1 mM spermidine. After 2 h incubation at 37 °C, RQ1 RNase-free DNase (Promega, Madison, WI, USA) was added to remove DNA template. The generated ssRNA was extracted using chloroform (Merck, Kenilworth, NJ, USA) and precipitated with 4 M LiCl (Merck, Darmstadt, Germany). The pellet was washed with 70% ethanol, dissolved in 400 µL of mQ water and re-precipitated with 0.3 M sodium acetate pH 6.5 and 67% EtOH. The resulting pellet was washed two times with 70% ethanol and dissolved in sterile mQ water.

UNAFold web server was used to predict the secondary structure of RNA molecules based on their sequence [43].

To produce HetRV6-specific dsRNA template for transcription reaction, HetRV6 RNA1^+^ was replicated using HetRV6 RdRp under optimal conditions (see Section 2.5). Then the produced dsRNA1 was fractionated with sequential LiCl precipitation as described in [44] to remove contaminating ssRNAs. The dsRNA was washed two times with 70% ethanol and dissolved in mQ water.

### 2.4. RNase Contamination Assay

The quality of the purified HetRV6 RdRp and its suitability for RNA polymerization assays was confirmed using RNase contamination assay. A total of 2 µg of enzymatically-produced HetRV6 RNA1^+^ (see Section 2.3) was incubated with different amounts of recombinant HetRV6 RdRp in the reaction buffer (see Section 2.5) for 1 h at 37 °C. The RNA integrity was verified by agarose gel electrophoresis.

### 2.5. Polymerase and Terminal Nulceotidyl Transferase Assays

The buffer composition for the polymerase assay was optimized in a series of experiments, where the concentration of individual buffer components was varied systematically (Figure S1). The dsRNA produced in the course of these experiments was fractionated using LiCl precipitation as described in [44]. The amount of the recovered dsRNA was measured with NanoDrop 2000c (Thermo Fisher Scientific, Carlsbad, CA, USA) to identify the conditions optimal for dsRNA synthesis. After the procedure of optimization, the polymerase activity of recombinant HetRV6 RdRp was typically assayed in a 20 µL reaction mixture containing 50 mM Hepes-KOH pH 8.0, 40 mM ammonium acetate (NH4OAc), 6% (*w*/*v*) polyethylene glycole 4000 (PEG4000), 5 mM MgCl_2_, 2 mM MnCl_2_, 0.1 mM EDTA, 0.1% Triton X-100, 0.2–1 mM of each nucleotide triphosphate (Thermo Fisher Scientific, Carlsbad, CA, USA), 70–140 pM ssRNA, and 0.8 U/µL RNasin (Promega, Madison, WI, USA). For the identification of newly synthesized RNA, the mixture was supplemented with 0.25 mCi/mL of [α-^33^P]-UTP (PerkinElmer, 3000 Ci/mmol). To study transcription mechanism, 90 nM of HetRV6 dsRNA1 was used as a template in polymerase reaction. All other components were kept the same. The TNTase activity was measured in the presence of only a single NTP type which was either 0.3 µM UTP supplemented with 0.25 mCi/mL of [α-^33^P]-UTP (PerkinElmer, 3000 Ci/mmol) or the same amounts of ATP and [γ-^33^P]-ATP (PerkinElmer, 3000 Ci/mmol). Reactions were initiated by addition of the HetRV6 RdRp to a final concentration of 0.8–1.2 μM. In the control reactions, enzyme was replaced with an equal volume of the RdRp storage buffer (see Section 2.2). The mixtures were incubated at 30 °C for 90–120 min, unless otherwise stated. All the experiments were repeated at least twice.

### 2.6. Analysis of the Reaction Products by Agarose Gel Electrophoresis and Autoradiography

A standard native 1% agarose gel containing 0.25 µg/mL EtdBr and buffered with Tris/borate/EDTA (TBE) buffer (50 mM Tris-borate pH 8.3, 1 mM EDTA) was used for electrophoretic analysis of RNA species. To this end, the reactions were stopped by addition of one reaction volume of 2 × U buffer containing 8 M urea, 10 mM EDTA, 0.2% SDS, 6% (*v*/*v*) glycerol, 0.05% bromophenol blue and 0.05% xylene cyanol FF [45]. To induce separation of dsRNA strands, 10 µL of 2 × U buffer was added to a 10 µL reaction sample followed by 5 min boiling. Then the samples were plunged into ice and incubated 20 min before analysis by agarose gel electrophoresis. After electrophoresis (5 V/cm), gels were photographed, dried, and exposed against the imaging plates (Fujifilm, Tokyo, Japan) for a suitable time depending on the activity of samples. The imaging plates were scanned with Typhoon TRIO Imager (GE Healthcare, Chicago, IL, USA). Open-source software suit Fiji [46] was used to compare density (intensity) of bands using a procedure described at https://lukemiller.org/index.php/2010/11/analyzing-gels-and-western-blots-with-image-j/ accessed on 5 May 2021. Origin 2020 (OriginLab Corporation, Northampton, MA, USA) was used to plot the data.

## 3. Results

### 3.1. Production of Recombinant Viral RdRp for Enzymatic Assays. HetRV6 RdRp Is an Active Replicase In Vitro

**Figure 1:**
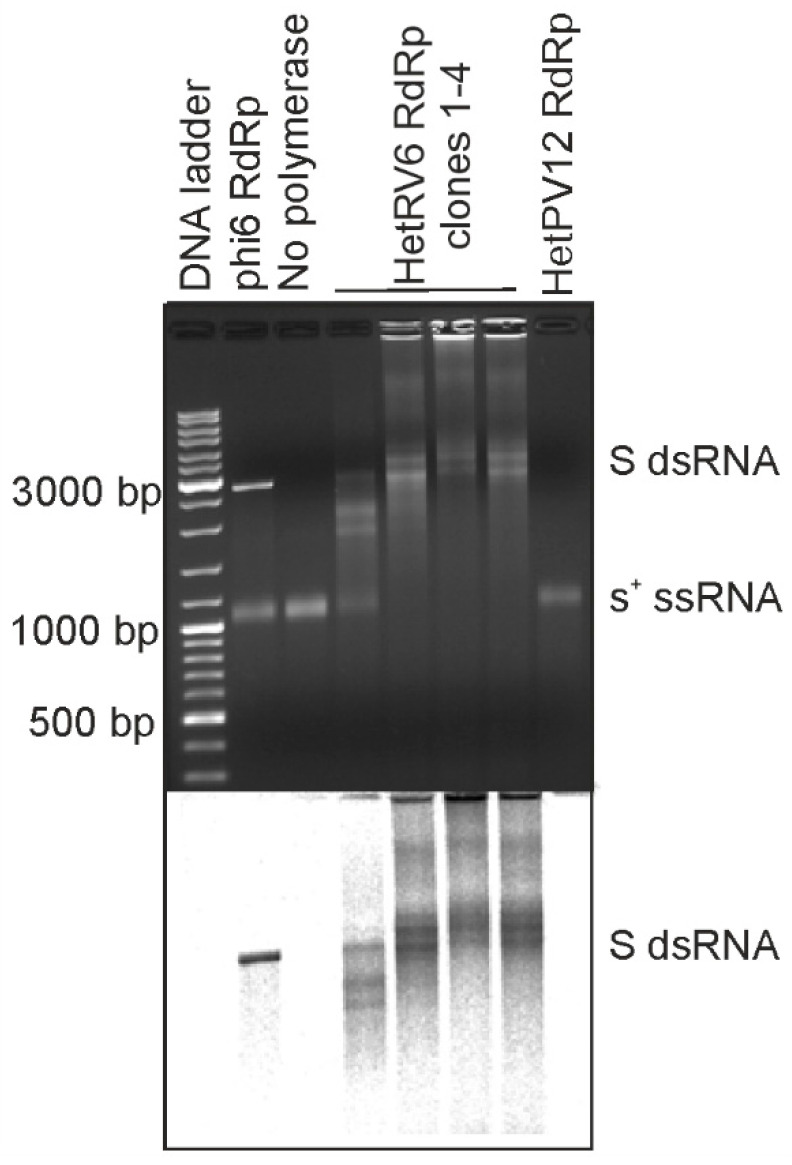
RNA synthesis directed by N-terminally His-tagged HetRV6 RdRp on phi6-specific template. Four different batches of N-terminally His-tagged HetRV6 RdRp were tested (clones 1–4) as well as similarly purified fractions from *E. coli* expression of N-terminally His-tagged HetPV12 RdRp. Full-length (+)RNA originating from the S genome segment of bacteriophage phi6 (s^+^) was used as a template. As a positive control, RdRp from bacteriophage phi6 was used. The reaction products were analyzed by native agarose gel electrophoresis using 0.8% agarose gel (top). The produced radiolabeled RNA molecules are visualized by autoradiography (below). The lengths of selected DNA ladder fragments are indicated on the left. The mobility of the template ssRNA and the dsRNA product are indicated on the right.

Initially we set out to produce recombinant RdRps from bisegmented dsRNA viruses commonly isolated from *Heterobasidion* species complex, i.e., the representatives of the family *Curvulaviridae* and *Patitiviridae*. To this end, we cloned RdRp genes from orthocurvulavirus HetRV6 and alphapartitivirus HetPV12 into pET-32b(+) plasmid for expression of the N-terminally His-tagged RdRps in *E. coli.* This allowed us to quickly evaluate the feasibility of obtaining soluble and active viral RdRps. Both RdRps were expressed in *E. coli*, but the solubility of the produced HetPV12 RdRp was clearly compromised (Figure S2A). We purified the cleared bacterial lysates using a column packed with Ni sepharose (see Section 2.2 and Figure S2B) and combined the fractions with the highest contents of the HetRV6 RdRp. We also combined the corresponding fractions from HetPV12 RdRp purification (Figure S2B), although the presence of the RdRp in these fractions could not be verified. To confirm the activity of the isolated RdRps, we performed polymerase assays (see Section 2.5) using bacteriophage phi6 S-segment specific s^+^ ssRNA as a template. Phi6 RdRp was included as a positive control of dsRNA synthesis [18,19]. A reaction, where RdRp was replaced with buffer, served as a negative control (Figure 1). Based on the mobility of the reaction products in native agarose gel, production of dsRNA, i.e., synthesis of a complementary (-)RNA strand for a given (+)RNA, was confirmed (Figure 1) for the N-terminally His-tagged HetRV6 RdRp. All preparations of HetRV6 RdRp produced from different bacterial clones were active in vitro and replicated the tested phi6-specific (+)RNA template. The similarly purified fractions originating from cells expressing mostly insoluble HetPV12 RdRp did not show any polymerase activity. This confirms that the RNA synthesis observed with the N-terminally His-tagged HetRV6 RdRp is an inherent property of the RdRp, and does not originate from co-purified bacterial proteins.

After confirmation that N-terminally His-tagged HetRV6 RdRp is an active replicase, we prepared a construct for the RdRp expression without any tags since we wanted to study the enzyme as close to its native state as possible, and cleavage of His-tag with enterokinase was not efficient. Strain BL21(DE3)pLysS(pET28a-HetRV6pol) produced detectable amount of soluble, approximately 70-kDa protein at 17 °C (Figure S3A; calculated molecular weight of the HetRV6 RdRp is 69.3 kDa). To separate the recombinant HetRV6 RdRp from bacterial proteins, resins routinely used for purification of enzymes involved in nucleic acid metabolism were tested. HetRV6 RdRp was bound to heparin agarose and anion exchange columns and eluted at ~400 mM NaCl (Figure S3A) and ~300 mM NaCl (Figure S3B), respectively. Subsequent elution from the gel-filtration column produced a single main peak (Figure S3C).

We compared the HetRV6 RdRp elution from the gel filtration column to the elution behavior of two other proteins of the similar size, namely bovine serum albumin (BSA; 66.5 KDa) and phi6 RdRp (74.8 KDa, see Figure S4). HetRV6 RdRp elution volume was comparable to that of BSA monomer suggesting a monomeric form for an active HetRV6 RdRp in solution. Retention of phi6 RdRp in gel filtration column corresponded to a 45 KDa protein [18]. Accordingly, we observed later elution of phi6 RdRp likely reflecting a more compact structure of the phi6 RdRp compared to HetRV6 RdRp. SDS-PAGE and western blot analyses confirmed the purity and identity of the purified HetRV6 RdRp (Figure S3C,D). The estimated yield of the purified protein was ~0.5 mg per liter of the bacterial culture. The produced protein did not contain contaminating RNases (Figure S3E). The HetRV6 RdRp without a His-tag was used in all subsequent experiments.

### 3.2. HetRV6 RdRp Initiates De Novo RNA Synthesis on Native and Heterologous Templates

**Figure 2:**
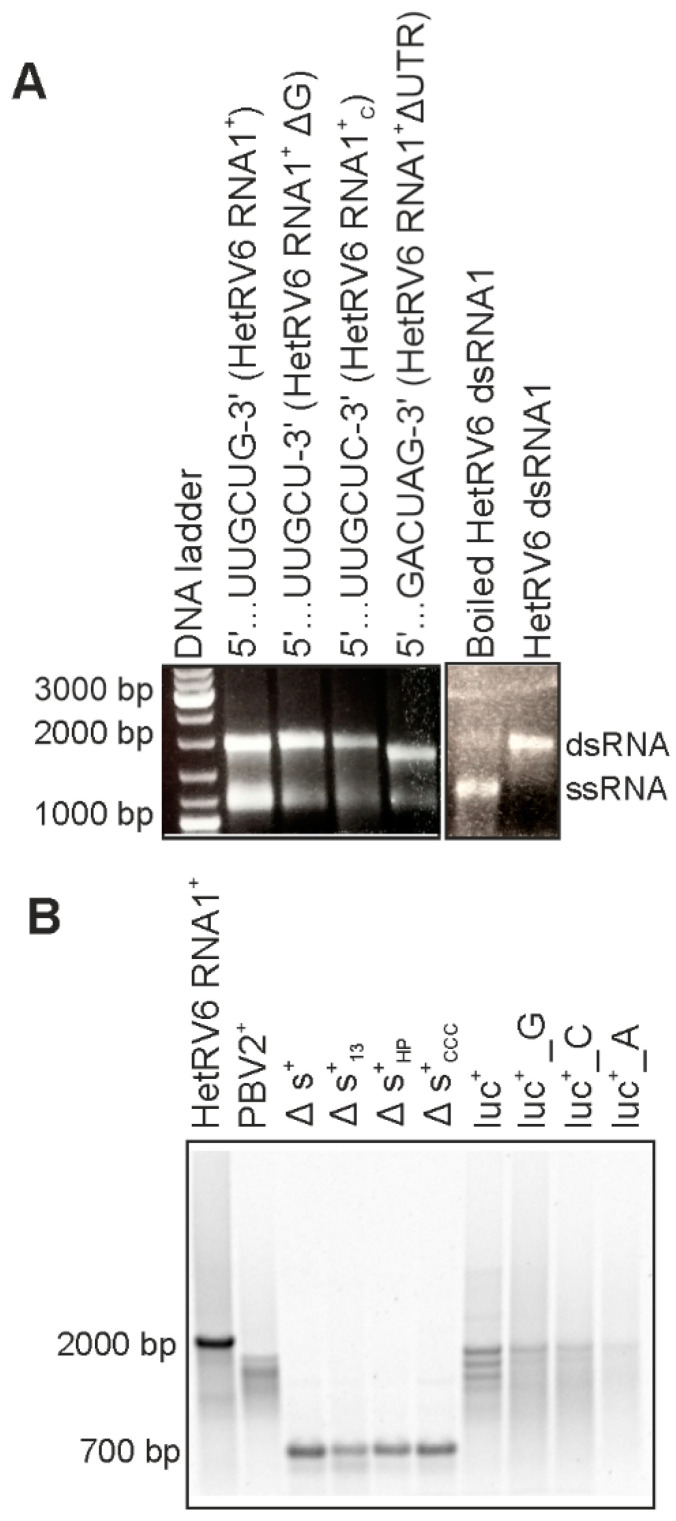
HetRV6 RdRp activity on native and heterologous templates. (**A**) Full-length HetRV6 RNA1^+^ and its modifications were used as templates in RNA polymerization reactions. The reaction products were analyzed by native agarose gel. The effect of boiling on the synthesized HetRV6 dsRNA1 reaction products is shown on the right. (**B**) Heterologous (+)RNA templates of viral and non-viral origin with variable 3′-ends were used as templates (refer to Table 1 for details). Native HetRV6 RNA1^+^ template was included as a control. The reaction products were analyzed by electrophoresis in native agarose gel, and corresponding autoradiogram is shown.

In order to maintain the genome integrity, the initiation of (-)RNA synthesis by dsRNA virus RdRp occurs at the very 3′-terminus of the (+)RNA strand. The HetRV6 RdRp synthesized (-)RNA on the native and heterologous (+)RNA templates with variable degree of efficiency (Figure 2). For most of the tested templates, full-length replication products were easily detected on native agarose gels using ethidium bromide staining (shown in Figure 1 and Figure 2A). The secondary structure of RNA1^+^ from HetRV6 was predicted using UNAFold software [43]. A characteristic 112-nt hairpin structure, potentially important for the RNA synthesis, was observed at the end of the 3′ untranslated region (3′ UTR) (Figures S5 and S6). Nevertheless, the HetRV6 RdRp retained its activity on the native ssRNA template even when the 3′-end was slightly modified, i.e., the G3′ was replaced with either U or C (Figure 2). Furthermore, the RNA synthesis was not compromised even when the entire 3′ and 5′ UTRs were deleted from the HetRV6 RNA1^+^ (RNA1^+^ΔUTR ssRNA). Hence, in vitro, no specific replication signal at the 3′ UTR or its interaction with 5′ UTR is required for the initiation of dsRNA synthesis by HetRV6 RdRp.

In addition to the native RNA1^+^ molecule, HetRV6 RdRp replicated a number of heterologous templates (Table 1, Figure 1 and Figure 2). However, the amounts of the synthetized dsRNA varied depending on the template sequence and the 3′-terminal fold of the RNA. Moreover, the production of full-length dsRNA was compromised in some cases, indicating reduced processivity. RNA2^+^ encoding RdRp from picobirnavirus (…CUGC3′ terminus) was not efficient template for HetRV6 RdRp. Contrarily, most tested batches of HetRV6 RdRp were active on phage phi6-specific ssRNA templates (Figure 1 and Figure 2; Table 1). Firefly luciferase mRNA (luc^+^; …AAGCUUA3′) and its variants luc^+^_G and luc^+^_C containing one nucleotide pyrimidine additions at the 3′-end (Table 1, Figure 2) were also accepted as templates. However, dsRNA product was hardly detectable even by autoradiography (Figure 2) when luc^+^_A ssRNA containing two adenines at the very 3′ terminus was used. This corroborates the previous observations made for RdRps from bacteriophages phi6 [40] and Qβ [47], hepatitis C virus (HCV) [48], and bovine viral diarrhea virus (BVDV) [49], which suggested that viral RdRps preferably initiate at pyrimidine-rich 3′ termini and two purines, especially adenines, in a row seems to adversely affect the initiation. However, other factors, such as the three-dimensional fold of the RNA [50], may also affect the template usage potentially explaining the relatively moderate activity observed on the picobirnavirus specific template. We did not observe correlation between the low processivity (Figure 1 and Figure 2) and overall percentages of different nucleotides in the template (Table S2) suggesting that local sequence and secondary structures of the template are more important factors for elongation than the nucleotide composition.

An internally truncated version of phi6 s^+^ ssRNA, Δs^+^ (Table 1), and its 3′-terminal modifications, Δs^+^_13_ and Δs^+^_HP_, were used to further study the specificity and impact of 3′-terminal RNA secondary structures on HetRV6 RdRp. The Δs^+^ ssRNA, similar to the wild-type s^+^, has a 3′-terminal fold with 5-nt long single-stranded extension at the very 3′-end. This template was suitable for dsRNA production catalyzed by HetRV6 RdRp. Δs^+^_13_ contains a 13 nt extension …CUAGAGGAUCCCC-3′, which can fold into a transient hairpin structure, while Δs^+^_HP_ harbors a stable tetraloop at the 3′-end originating from the sequence …GUAGGGGUUCGCCCC-3′ (the residues forming the predicted stem of the hairpin loop are underlined [40]). In addition, Δs^+^_CCC_ ssRNA containing triple cytosine at the 3′-terminus of Δs^+^ was used. Δs^+^_13_ and Δs^+^_HP_ templates were replicated with HetRV6 RdRp, although less efficiently than Δs^+^ and Δs^+^_CCC_ containing free 3′-ends (Figure 2).

The RdRps of RNA viruses can initiate RNA synthesis either de novo or using nucleic acid-primed, protein-primed or back-primed mode [1]. To differentiate between de novo and back-primed initiation we boiled HetRV6 dsRNA1 synthesized by the HetRV6 RdRp and analyzed the reaction product by agarose gel electrophoresis (see Section 2.6 and Figure 2A). The boiling resulted in dissociation of dsRNA molecules indicating that (+) and (-) strands are not covalently bound and initiation occurs de novo. Furthermore, ∆s^+^_13_ and ∆s^+^_HP_ templates, which 3′-ends can fold into loop structures that facilitate initiation by back-priming [40], were not preferred templates for HetRV6 RdRp as a higher RNA synthesis activity was detected with the ∆s^+^ template which has 5-nt-long single-stranded 3′-end (Figure 2B). Thus, initiation from the end of a linear template molecule rather than from a preformed loop structure that mimics template-primer complex is preferred by the HetRV6 RdRp.

### 3.3. HetRV6 RdRp Displays Slow RNA Polymerization Rate In Vitro

**Figure 3:**
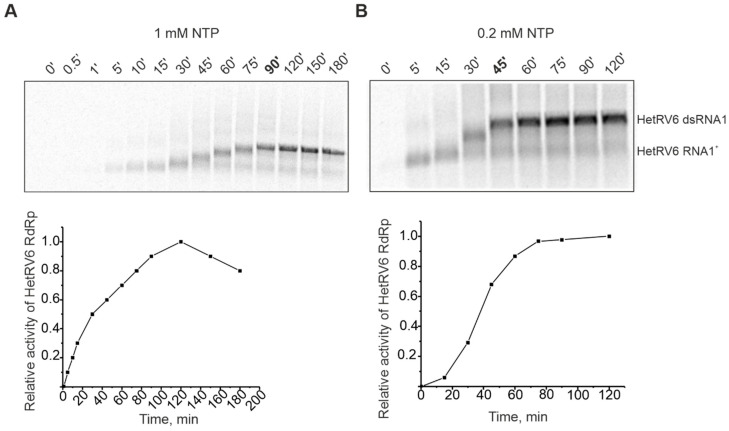
Time course of dsRNA synthesis catalyzed by HetRV6 RdRp in the presence of 1 mM (**A**) and 0.2 mM (**B**) NTPs. A 150-µL polymerase reaction containing HetRV6 RNA1^+^ was incubated at 30 °C in the presence of HetRV6 RdRp. Five microliter aliquots, sampled at the indicated time points, were analyzed by agarose gel electrophoresis followed by autoradiography. Representative autoradiography images of the sample analysis (upper panel) and graphical presentation of the same data (lower panels) are shown. Quantitation of the full-length dsRNA product was done by densitometry with Fiji image analysis software (see Section 2.6). The data were normalized so that the highest observed value for the density of dsRNA band on each panel was set to 1.0. The mobility of the template HetRV6 RNA1^+^ and the dsRNA product are indicated on the right.

Previously studied viral RdRps are mainly from human pathogens or bacterial viruses which replicate relatively fast. HetRV6 is a representative of an asymptomatic fungal virus and, thus, comparison of its RdRp polymerization rate to other studied viral RdRps is of biological interest. Time-course experiments were carried out to follow the synthesis of ^33^P-labeled RNA species, and the incorporation of [α-^33^P]UTP was detected by autoradiography. The full-length HetRV6 dsRNA1 was synthesized in 90 min at 30 °C, when 1 mM of each NTP was present in the reaction mixture (Figure 3A), i.e., RNA chain polymerization rate was about 23 nt per min. When the concentration of ribonucleotides was reduced to 0.2 mM, the polymerization rate increased and full-length product was observed on the gel already after 45 min, i.e., about 46 nt was incorporated into nascent RNA chain in a minute (Figure 3B). Interestingly, the kinetics of accumulation of radiolabeled nucleic acid was faster when 1 mM NTPs were used, but this did not result in more rapid synthesis of the full-length dsRNA (compare curves A and B in the Figure 3). Low NTP concentrations also favored minus strand synthesis in phi6 procapsids [51].

The replication reaction occurred at the room temperature as well, but at a slower rate (Figure S7). Temperature increase to 37 °C resulted in substantial rise of the replication rate, and even at 1 mM NTPs a full-length dsRNA product accumulated by the first hour of incubation (Figure S7). However, such high temperature is not natural for the HetRV6 life cycle, and we did not use it for subsequent experiments.

### 3.4. Effect of NTP Imbalance and NTP Modifications on HetRV6 RdRp Activity

**Figure 4:**
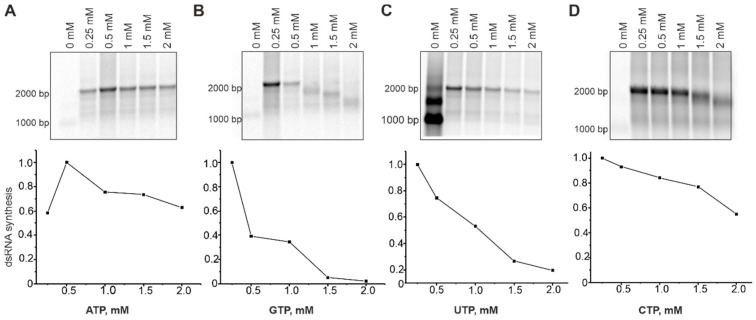
Effect of the NTP concentrations on dsRNA synthesis by HetRV6 RdRp. In a standard polymerase assay with a native RNA1^+^ template, concentration of ATP (**A**), GTP (**B**), UTP (**C**), or CTP (**D**) varied in the range of 0 to 2 mM, while the concentration of other three NTPs was kept at 0.2 mM. After 1.5 h incubation at 30 °C, the reactions were terminated by adding equal volume of 2 × U buffer (see Section 2.6). Reaction products were analyzed by agarose gel electrophoresis and radioautography (top). The densitometric analysis of the gels using Fiji software shows the density (intensity) of the bands corresponding to the both full-length and truncated dsRNA products. The data were normalized so that value 1.0 was assigned to the band with the highest density.

To better understand the role of individual NTPs in dsRNA synthesis and identify their optimal concentrations, we set up polymerase assays containing a native RNA1^+^ template (Table 1 and Table S2), 0.2 mM of three NTPs, and a variable concentration (0–2 mM) of a selected NTP (Figure 4). Notably, concentrations of GTP larger than 0.5 mM, and CTP larger than 1 mM prevented synthesis of full-length dsRNA molecules. The content of CMP and GMP in the synthesized RNA strand is slightly higher compared to AMP and UMP (Tabe S2). However, the increased concentrations of their precursors negatively affected dsRNA polymerization. We believe that imbalance in NTP concentrations might increase nucleotide misincorporations resulting in polymerase pausing or even stalling on the template [52]. Optimal concentrations for GTP, UTP, and CTP were between 0.2 and 0.25 mM, while optimum for ATP was 0.5 mM. Moreover, higher concentrations of ATP (1–2 mM) enhanced dsRNA synthesis (Figure 4). ATP is the second NTP, which is incorporated into the growing RNA strand and its increased concentration might facilitate the formation of the initiation complex.

**Figure 5:**
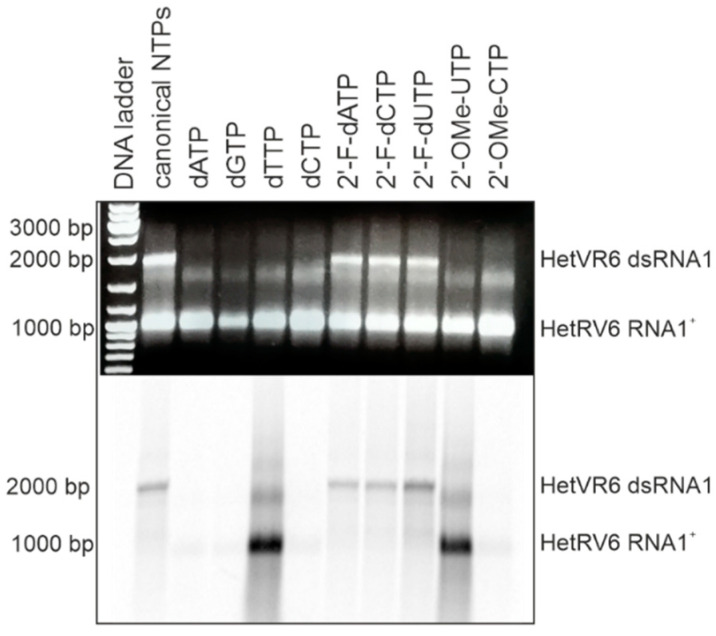
HetRV6 RdRp ability to use modified NTPs as a substrate for the RNA-directed synthesis of a (-)RNA strand. One of the NTPs in polymerase reaction was replaced by its modified analogue, and a standard polymerase reaction with [α-^33^P]-UTP was incubated 1.5 h at 30 °C. Native agarose gel is on the top, and autoradiogram of the same gel is shown at the bottom. The mobility of the template HetRV6 RNA1^+^ and the dsRNA1 product are indicated on the right.

In addition to canonical NTPs, HetRV6 RdRp utilized 2′-fluoro modified NTPs for dsRNA synthesis with similar efficiency (Figure 5). However, NTPs containing larger modifications at the 2′ position of ribose ring (e.g., methoxy group) or canonical dNTPs were not incorporated in the nascent (-)RNA strand. Since we used [α-^33^P]-UTP to visualize polymerase reaction products, this label was incorporated into the growing chain in the absence of canonical UTP or when UTP was replaced by either dTTP or 2′-OME-UTP, which resulted in heavily labeled reaction products visible on the autoradiogram. However, the reaction stalled when [α-^33^P]-UTP substrate was consumed, and no full-length dsRNA was observed (see Figure 4 and Figure 5).

### 3.5. HetRV6 RdRp Replicase Activity Is Dependent on Mn^2+^ Cations but Inhibited by Ca^2+^ Ions

**Figure 6:**
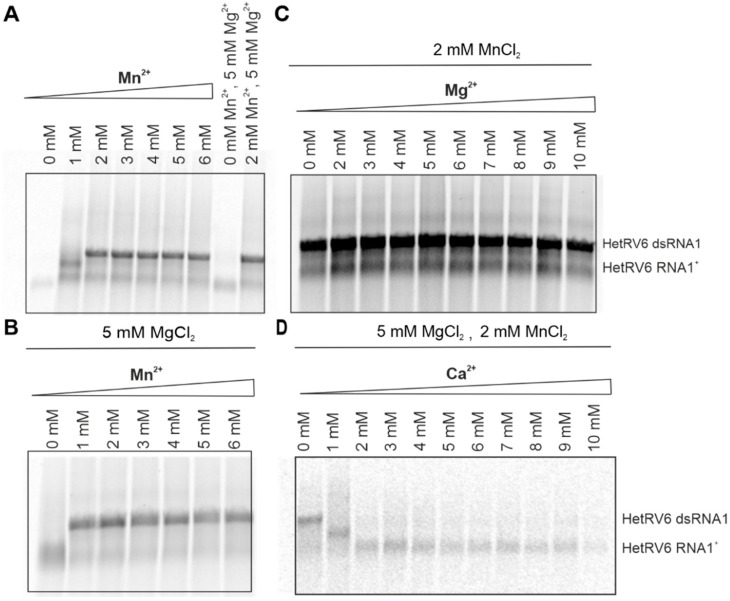
Role of divalent cations in dsRNA synthesis catalyzed by HetRV6 RdRp. (**A**) Buffer for the polymerase assays was prepared without addition of any divalent cations. Mn^2+^ was added to a final concentration of 1–6 mM. (**B**) Standard polymerase assays were supplemented with 5 mM MgCl_2_ and included variable concentration of Mn^2+^. (**C**) In the reactions containing 0−10 mM Mg^2+^, 2 mM MnCl_2_ was added. (**D**) Reactions with 0−10 mM Ca^2+^ contained both 5 mM MgCl_2_ and 2 mM MnCl_2_. The polymerase assays were incubated at 30 °C during 1.5 h, and then analyzed on native agarose gel followed by autoradiography. The mobility of the template HetRV6 RNA1^+^ and the dsRNA1 product are indicated on the right.

Although Mg^2+^ ions are typically involved in the catalytic function of viral RdRps, Mn^2+^ can stimulate RNA polymerization in a number of RNA viruses including phi6 [9,18] and related phages phi8, phi12, phi13 [20,53], classical swine fever virus [54], BVDV [55], HCV [56], GB virus-B [57], and infectious pancreatic necrosis virus (IPNV) [58]. For better understanding of the role of divalent cations in HetRV6 RdRp-catalyzed RNA synthesis, we kept the same basic buffer composition as described in 2.5, and systematically changed the concentration of a single divalent cation. Mn^2+^ was indispensable for dsRNA synthesis, and its optimal concentration was within 2–4 mM (Figure 6A,B and Figure S1). Although dsRNA synthesis was further stimulated in the presence of 1–5 mM Mg^2+^ (Figure 6C and Figure S1), Mn^2+^ alone was sufficient to catalyze the reaction. However, at Mn^2+^ concentrations below 2 mM, no synthesis of the full-length dsRNA product was detected (Figure 6A).

Ca^2+^ inhibited elongation of a growing (–)RNA chain at concentrations above 1 mM despite the presence of the other divalent cations in the reaction mixture. Even at 1 mM Ca^2+^, we did not observe synthesis of a full dsRNA product (Figure 6D).

### 3.6. HetRV6 RdRp Posseses Terminal Nuleotidyl Transferase (TNTase) Activity

**Figure 7:**
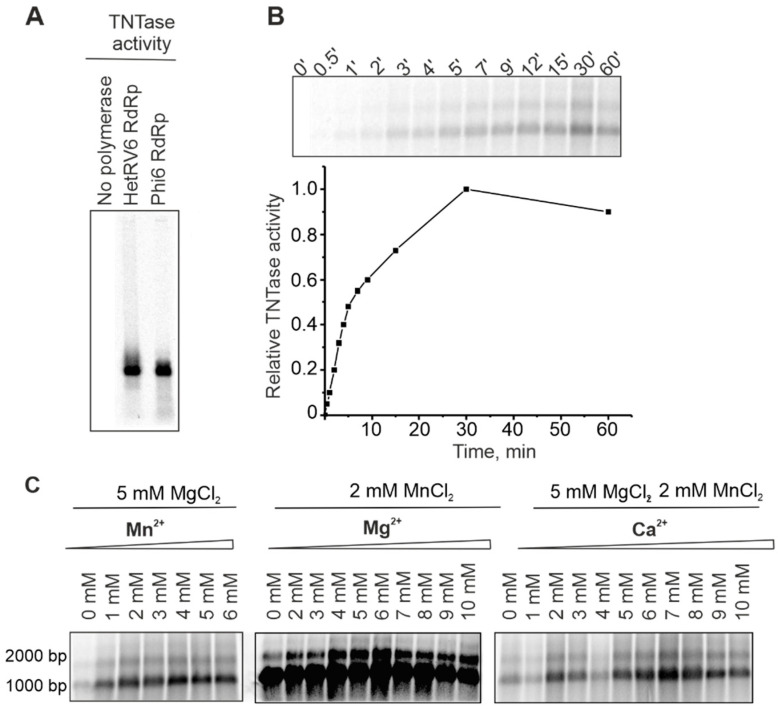
TNTase activity of HetRV6 RdRp. (**A**) Δs^+^ ssRNA was used as a template in the TNTase assays catalyzed either by phi6 RdRp or HetRV6 RdRp. Autoradiogram of the reaction products after agarose gel electrophoresis shows label incorporation from [α-^33^P]-UTP. (**B**) A time course of the TNTase activity on the viral HetRV6 RNA1^+^. Quantitation of the labelled RNA product is shown below the autoradiogram of the gel. (**C**) Influence of divalent cations on TNTase activity. Viral HetRV6 RNA1^+^ was used as a template and UTP as a substrate (no other NTPs were included).

A number of viral RNA polymerases catalyze template-independent addition of one or several ribonucleotides to the 3′-end of an RNA molecule in a reaction which is referred to as terminal nucleotidyl transfer [21,59,60,61]. In order to detect potential TNTase activity, we set up reactions with a single NTP, either UTP or ATP (see Section 2.5). Similar to phi6 RdRp [59], HetRV6 RdRp catalyzed incorporation of a label from [α-^33^P]-UTP to the ssRNA molecules used as templates (Figure 7A). Label incorporation from [γ-^33^P]ATP was not detected (Figure S8), which indicates that HetRV6 RdRp catalysed extension of the given ssRNA substrate from its 3′-end rather than ligation of NTP to the 5′-end.

In the HetRV6 RdRp catalyzed TNTase reaction, labeled RNA1^+^ appeared after 0.5 min and plateau was reached in approximately 30 min (Figure 7B). Both isoforms of HetRV6 RNA1^+^ having different electrophoretic mobilities on the gel became labeled. TNTase activity was greatly impared in the absence of Mn^2+^, and did not change significantly when Mn^2+^ was added in the concentration range of 2–6 mM (Figure 7C). Mg^2+^ was not required for TNTase activity, but the amount of incorporated label greatly increased when Mg^2+^ was added in 4–8 mM concentration (Figure 7C). Ca^2+^ concentrations of up to 10 mM did not inhibit this type of activity (Figure 7C).

### 3.7. HetRV6 RdRp Demonstartes Semi-Conservative Mechanism of Transcription

**Figure 8:**
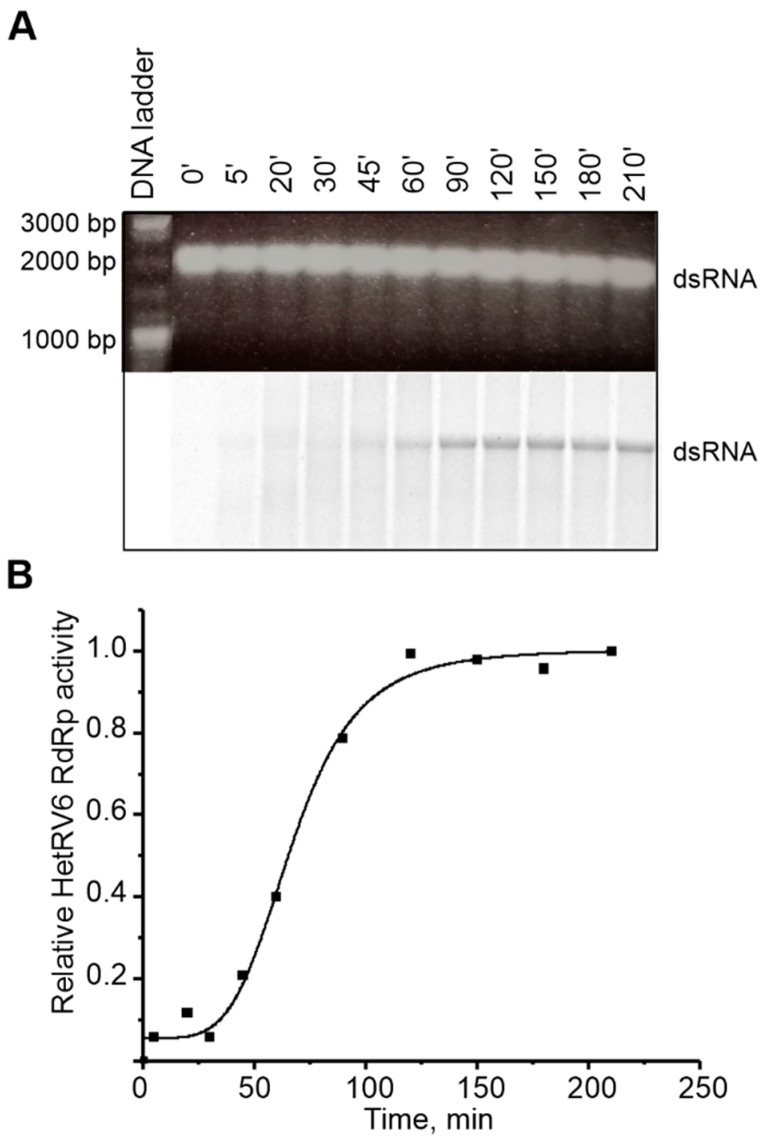
Transcription activity of HetRV6 RdRp. (**A**) HetRV6 dsRNA1 was used as a template in the polymerase reaction. A 10 µL aliquot was removed from the reaction at the indicated time point. The native agarose gel is presented at the top. A corresponding autoradiogram shows label incorporation from [α-^33^P]-UTP (below). (**B**) Time course of the transcription activity on the viral HetRV6 dsRNA1. The intensity of the labeled product in (**A**) was measured using Fiji software, and the data were normalized so that the value 1.0 was attributed to the highest value of intensity.

Viruses with dsRNA genomes utilize both conservative (members of the families *Reoviridae* and *Totiviridae*) and semiconservative (families *Cystoviridae*, *Partitiviridae*, *Birnaviridae*, and *Picobirnaviridae*) mechanism of transcription. The conservative mode is characterized by production of a nascent ssRNA strand on a dsRNA template via local melting of dsRNA at the site of interaction with RdRp leaving the rest of the template dsRNA intact. In the process of semi-conservative transcription, a growing chain displaces the parental positive strand from the dsRNA template. To determine the transcription mechanism used by HetRV6, we established a polymerase reaction which included HetRV6 dsRNA1 as a template. If transcription occurs semi-conservatively, and it is carried out in the presence of [α-^33^P]-UTP we would expect release of unlabeled (+)RNA strand at the end of the first round of transcription and label incorporation to the (–) strand of the dsRNA. After 45 min reaction, the dsRNA1 became labeled, and the label intensity was increasing within two hours (Figure 8A,B). We did not observe any labeled ssRNA molecules, which supports the semi-conservative mechanism of transcription. The TNTase activity cannot be an explanation for the observed phenomenon since it is not registered in the presence of all NTPs in the concentration required for polymerase reaction. Nevertheless, the transcription reaction in the absence of other viral proteins is not very efficient and most probably ends after a single round.

## 4. Discussion

In the present study, we characterized the activities of RdRp from the recently taxonomically assigned mycovirus HetRV6. We showed that the isolated recombinant HetRV6 RdRp catalyze nucleotidyl transfer in both template-dependent and template-independent manner (Figure 1, Figure 2 and Figure 7), we determined optimal conditions for the catalysis, such as temperature, buffer pH, concentration of salts, NTPs, and divalent cations, optimal ratio of enzyme and RNA template (Figure 4 and Figure 6, Figures S1 and S6). Furthermore, our results suggest a semi-conservative mechanism of transcription for HetRV6 (Figure 8).

The RdRp from dsRNA viruses catalyze both synthesis of a (-)RNA on the (+)RNA template and production of a (+)RNA strand on the dsRNA template. The former activity is generally referred to as replication, and the latter as transcription. Recombinant HetRV6 RdRp can efficiently replicate ssRNA templates in vitro (Figure 1 and Figure 2) and does not require other viral or/and host proteins for its activity. Notably, not all isolated viral RdRps are active in vitro, and RdRps of dsRNA viruses might be inactive in the absence of their capsid proteins. For instance, rotavirus RdRp VP1 requires inner capsid protein VP2 for its activity. Although isolated VP1 can bind NTPs [62] and ssRNA [63], the interactions are weak to provide efficient RNA synthesis. A number of recombinantly produced viral RdRps retain some polymerase activity when overexpressed, but the activity is often weak and can be detected only with short templates using labeling of the synthesized RNA strands [64,65,66]. Instead, the recombinant HetRV6 RdRp displayed strong full-template length RNA synthesis activity on multiple different templates that varied in size from approximately 700 bp to 3000 bp (Figure 1 and Figure 2; Table 1).

Our results suggest that HetRV6 RdRp initiates ssRNA replication de novo, i.e., it does not require primer or a template enabling back-priming for this activity (see Section 3.2 and Figure 2). Primer-independent initiation has been described for most of dsRNA viruses. However, there are some exceptions, such as dsRNA viruses from the family *Birnaviridae*, which use self-priming, i.e., the RdRp functions as a protein-primer [58,67]. Rotaviruses (*Reoviridae*) can synthesize a 5′-end phosphorylated dinucleotide pGpG or ppGpG, which is then apparently used as a short primer to produce a full-length RNA product [68].

The polymerization rate of a (-)RNA synthesis catalyzed by HetRV6 RdRp is about 46 nt/min under optimal conditions, which is rather slow compared to other previously described RdRps. For example, phi6 RdRp incorporates 7200 nt/min at 28 °C [18] while the elongation rates of picornaviral RdRps range from 5280 nt/min (poliovirus) to 1320 nt/min (coxsackievirus B3) at 37 °C [69]. The rate of transcription by human PBV RdRp, which is generally less efficient than replication in vitro, is 210 nt/min at 37 °C [21]. We hypothesize that the low polymerization rate of HetRV6 RdRp and, consequently, the slow replication of the virus might be related to the usually cryptic manifestation of the infection [32].

HetRV6 RdRp demonstrated activity on variety of ssRNA templates (Figure 1 and Figure 2). Viral polymerase subunits, which are active in vitro on a wide range of heterologous templates, can be potentially used as a tool for molecular biology and biotechnology, as, for instance, polymerase from phi6 bacteriophage which is widely applied to produce dsRNA for RNAi applications [14,15,16,70,71]. Similar to the phi6 RdRp [14], HetRV6 RdRp can incorporate 2′-fluoro-modified NTPs and produce modified dsRNA molecules, which seem to be more potent mediators of RNAi [14]. These properties make HetRV6 polymerase a potential tool for dsRNA production for RNAi applications. The low RNA replication rate of HetRV6 RdRp likely only has a minor role in the selection of the most suitable candidate for dsRNA production as other factors, such as production cost, stability, fidelity, and utilization rate of modified NTPs, may be more important in the development of biotechnological tools based on viral RdRps.

The relative concentration of nucleotides is crucial for the optimal activity of HetRV6 RdRp. We discovered that elevated concentrations of UTP, CTP, and GTP in the reaction mixture, negatively affected replication, and their optimal concentration was 0.2−0.25 mM since a significant decrease in dsRNA yield was observed with higher NTP concentrations (Figure 4). We did not detect a synthesis of the full-length HetRV6 dsRNA1, when GTP concentration exceeded 0.5 mM and CTP–1 mM. At the same time, ATP at the concentration 0.5−1.5 mM stimulated dsRNA synthesis (Figure 4). According to the RNA sequence, the first nucleotide incorporating into the minus-sense growing chain of HetRV6 RNA should be cytosine and the second one is adenine. However, elevated concentration of the initiating CTP did not result in a better replicase activity of the HetRV6 RdRp. Conversely, the replicase activity of phi6 RdRp improves, when both ATP and GTP, the first two nucleotides to be incorporated into the nascent RNA chain, are present at 1 mM concentration [18,19].

In addition to NTPs, divalent metal ions considerably affected RNA synthesis, which is related to their paramount role in the catalysis of the phosphodiester bond formation. According to a two-metal-ion mechanism of nucleotide addition [6], two catalytically active divalent metal ions designated as A and B are required for the reaction. Ion A facilitates deprotonation of the hydroxyl group at the 3′-end of the growing polynucleotide chain to ensure nucleophilic attack on the α-phosphate of the incoming NTP substrate. Ion B binds the phosphates of the incoming NTP and assists the leaving of the pyrophosphate, and both metal ions stabilize the ternary complex intermediate. A third metal ion binding at approximately 6 Å distance from the catalytic site has been observed in a number of viral RdRps [7]. This binding site is often occupied by Mn^2+^ ion [7,72]. Our results showed that HetRV6 RdRp efficiently catalyzed incorporation of NMPs into the growing RNA chain in the presence of only Mn^2+^ ions, whereas Mg^2+^ was not absolutely required for its activity. However, Mg^2+^ ions did stimulate RNA synthesis. It has been demonstrated that non-catalytic Mn^2+^ ions are essential for the efficient template binding, initiation, and elongation via increasing structural flexibility of RdRp and establishing correct coordination of a catalytic aspartate needed to properly interact with the triphosphates of the incoming NTPs [9]. Furthermore, the transition from initiation to elongation is entirely dependent on the presence of Mn^2+^ ions in the reaction buffer reflecting structural changes that result in Mn^2+^ ion dissociation from the RdRp and the requirement to reload the binding site with Mn^2+^ to continue elongation [73]. Accordingly, the addition of Mn^2+^ ions to the Mg^2+^-containing reaction buffer stimulates the activity of a number of viral RdRps, including RdRp from phi6 and related bacteriophages, poliovirus, HCV, and reovirus [9,20,56,65,74]. Mn^2+^ also supports polymerization in the absence of Mg^2+^ ions suggesting that it can serve as a catalytic ion [9,56]. Mn^2+^ is very similar to Mg^2+^ in its physical properties, but it is more polarizable and binds more tightly to the carboxylates of Asp_A_ and Asp_C_, as well as to triphosphate moiety of NTPs [75], which can be the reason for it being more efficient in catalysis of the RNA polymerization directed by HetRV6 RdRp in vitro.

Concentrations of Ca^2+^ exceeding 1 mM inhibited polymerization despite the presence of both Mg^2+^ and Mn^2+^, meaning that Ca^2+^ can outcompete them in interaction with catalytic aspartate residues (Figure 6). Crystal structure of the initiation complex consisting of phi6 RdRp, GTP and Ca^2+^ showed that two Mg^2+^ catalytic ions can be indeed replaced with Ca^2+^. Ca^2+^ in the position of the catalytic ion B is displaced by 4.6 Å from the original location and does not participate in the coordination of phosphates of the incoming NTP [76]. Although the other Ca^2+^ retains the initial position of the catalytic ion A [76], this cation is not as efficient in reducing pKa value of the 3′-OH as Mn^2+^ and Mg^2+^. Furthermore, the ionic radii of magnesium and manganese are very similar, 0.86 and 0.81 Å, respectively, whereas the ionic radii of Ca^2+^ is significantly larger, 1.1 Å [77]. The ionic radii of metal ion A play an important role in determining the distance between 3′-OH group and α-phosphorous atom of the incoming NTP, which must be close enough to allow the covalent bond formation.

In addition to replication of (-)RNA, HetRV6 RdRp catalyzes transcription on dsRNA1 template to produce (+)RNA molecules. Similar to the most dsRNA viruses, with the exception of those from the family *Reoviridae* and some representatives of *Totiviridae*, HetRV6 RdRp utilizes semi-conservative mode of transcription, i.e., it displaces one of the dsRNA strands by a newly synthesized ssRNA chain. The semi-conservative mechanism was supported by the labeling of dsRNA in the polymerase reaction, whereas labeled (+)RNA was not detected (Figure 8). However, this reaction is less efficient than replication and does not continue after a single round of transcription, and, therefore, we could not detect labeled ssRNA products. Accordingly, no ssRNA products were detected when a semi-conservative transcription reaction catalyzed by isolated phi6 RdRp [19] or RdRp from human PBV [21], which highlights the importance of the other viral proteins in the repeated initiation of transcription observed within transcriptionally active dsRNA virus core particles [78].

TNTase activity was described for a number of (+)RNA viruses, including representatives of the families *Flaviridae* (HCV, BVDV), *Nodaviridae* (Wuhan nodavirus, flock house virus), *Caliciviridae* (sapovirus, norovirus), and *Picornaviridae* (poliovirus) [60,79,80,81,82]. Additionally, viruses with a dsRNA genome, namely bacteriophage phi6 (*Cystoviridae*) [59], human PBV (*Picobirnaviridae*) [21], and IPNV (*Birnaviridae*) [58] were reported to have TNTase activity. Here we showed that HetRV6 RdRp catalyzes template-independent addition of NTPs to the 3′-end of ssRNA. The role of TNTase activity for the virus life cycle is still elusive, but there is a hypothesis that it is useful to restore 3′-end initiation site that might degrade under attack of cellular RNases [60]. The TNTase activity might be responsible for the fact that HetRV6 RdRp retained activity on truncated templates. Nevertheless, it is important to analyze HetRV6 RdRp specificity for the incorporated NTPs and its dependence on the template secondary structure to understand the role of TNTase activity in the viral life cycle.

To sum up, our study is the first experimental proof that HetRV6 RdRp possesses replicase activity in vitro and is able to initiate a complementary strand synthesis on the native and heterologous ssRNA templates in a primer-independent manner. We also suggested semi-conservative mechanism of transcription for HetRV6. The catalytic activities are strongly dependent on the presence of Mn^2+^ cations. The ability of HetRV6 RdRp to include modified NTPs into a nascent RNA chain makes it a potential tool for biotechnological applications. The TNTase activity of the enzyme might be useful in terms of non-templated enzymatic RNA synthesis.

## Figures and Tables

**Table 1:** SsRNA templates used in the polymerase assays.

SsRNA Name	Plasmid Used for Preparation	3′-end Sequence; Description	Length, nt
HetRV6 RNA1^+^	pCR2.1-TOPO-HetRV6-RNA1 [32]	5′-…GCUG-3′; native HetRV6 RNA1^+^	2050
HetRV6 RNA1^+^ΔG	the same	5′-…GCU-3′; RNA1^+^ with 3′G deletion	2049
HetRV6 RNA1^+^G→C	the same	5′-…GCUC-3′; RNA1^+^ with 3′G substitution	2050
HetRV6 RNA1^+^ΔUTR	the same	5′-…CTAG-3′; RNA1^+^ with deletion of the UTRs ^a^	1821
PBV2^+^	pMA-RQ_PBV2 [21]	5′-…UAC-3′; native human PBV RNA2^+^	1745
phi6 s^+^	pLM659 [39]	5′-…CUCU-3′; native phi6 S-segment ssRNA	2948
phi6 ∆s^+^ (phi6 S-segment Δ593—2830)	pEM15 [19]	5′-…CUCU-3′; s^+^ with deletion of nts 593—2830	710
phi6 ∆s^+^_13_	the same	5′-…AUCCCC-3′; sΔ^+^ with a 13-nt-long extension forming a hairpin at the 3′-end	723
phi6 ∆s^+^_CCC_	the same	5′-…CUCUCUCCC-3′; ∆s^+^ with three nt extension at the 3′-end	713
phi6 ∆s^+^_HP_	pEM19 [40]	5′-…UUCGCCCC-3′; sΔ^+^ with a 13-nt stable tetraloop at the 3′-end	725
luc^+^	pT7luc [41]	5′…UUA-3′; firefly luciferase mRNA	1824
luc^+^_G	the same	5′…UUAG-3′; firefly luciferase with 3′G extension	1825
luc^+^_C	the same	5′…UUAC-3′; firefly luciferase with 3′C extension	1825
luc^+^_A	the same	5′…UUAA-3′; firefly luciferase with 3′A extension	1825

^a^ UTR, untranslated region.

## Data Availability

The data that support the findings of this study are available from the corresponding author upon reasonable request.

## Supplementary Materials

The following are available online at https://www.mdpi.com/article/10.3390/v13091738/s1, Figure S1: Optimization of the buffer composition for HetRV6 RdRp catalyzed polymerase reaction, Figure S2: Purification of RdRp of curvulavirus HetRV6 and partitivirus HetPV12, Figure S3: Purification of recombinant HetRV6 RdRp, Figure S4: Elution of HetRV6 RdRp, phi6 RdRp, and BSA from gel filtration column, Figure S5: Secondary structure prediction of HetRV6 RNA1^+^, Figure S6: Secondary structure of the 3′-end from HetRV6 RNA1^+^, Figure S7: Influence of temperature on the HetRV6 RdRp catalyzed RNA replication rate, Figure S8: R TNTase activity of HetRV6 RdRp, Table S1: Oligonucleotides used in the study, Table S2: Percentages of different nucleotides in the templates ssRNAs.
