# Supplementary material for: RNA-Dependent RNA Polymerase from Heterobasidion RNA Virus 6 Is an Active Replicase In Vitro
Source: Viruses. 2021 Aug 31;13(9):1738. doi: 10.3390/v13091738 (PMC8473416; doi:10.3390/v13091738)
Supplement: Supplementary file 1 [file viruses-13-01738-s001.zip › viruses-1342797-supplementary.pdf]

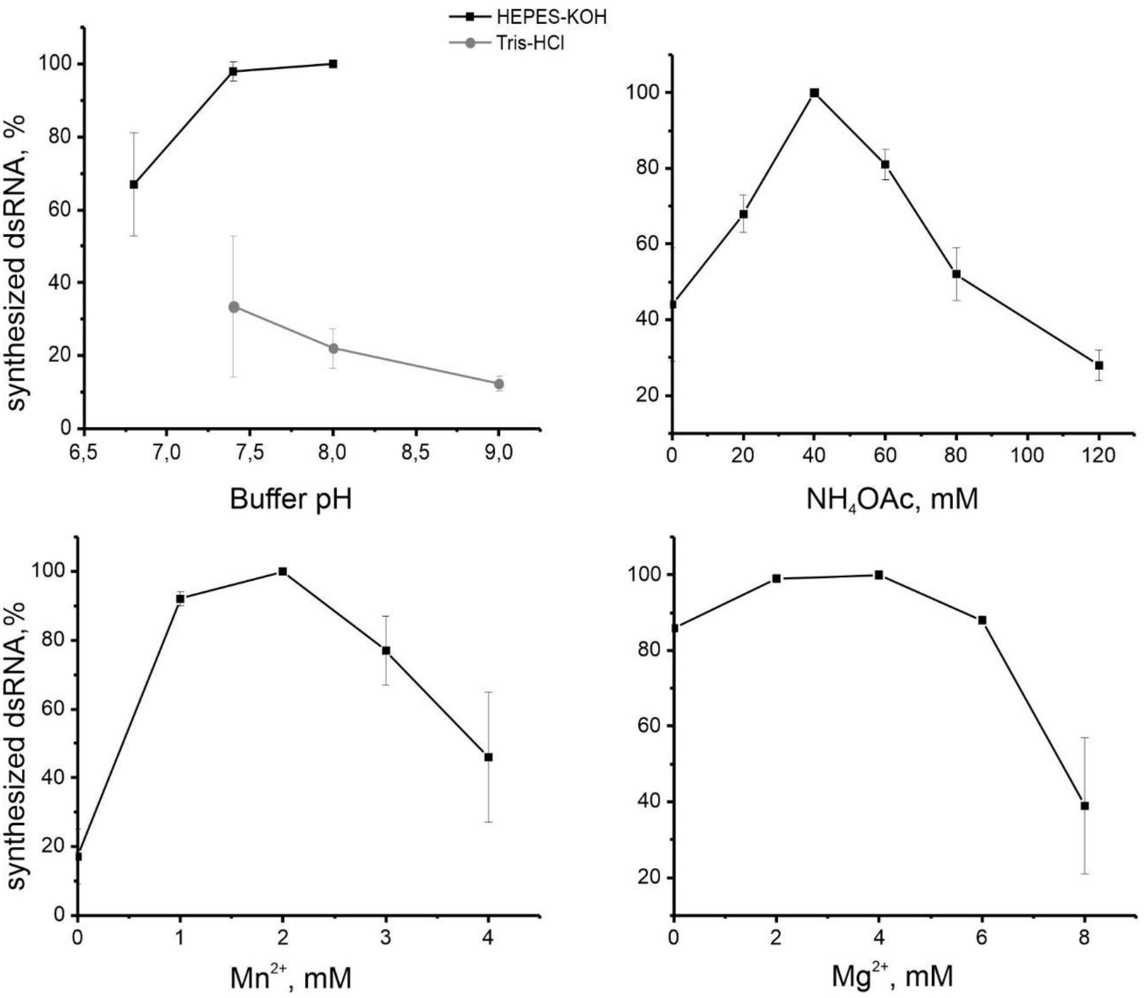

Figure S1. Optimization of the buffer composition for HetRV6 RdRp catalyzed polymerase reaction. Initially buffer containing 50 mM Tris-HCl pH 8.9, 80 mM ammonium acetate (NH4OAc), 6% (w/v) PEG4000, 5 mM $MgCl_2$, 1 mM $MnCl_2$, 0.1 mM EDTA, 0.1% Triton X-100, 1 mM of each nucleotide triphosphate [18] was used. pH and salt concentrations were systematically varied to identify the optimal buffer content. After 2 h incubation of the reactions at 30°C, the molecules of ssRNA were fractionated by 2 M LiCl (Merck) followed by the precipitation of dsRNA from the resultant supernatant with 4 M LiCl. The pellets were dissolved in 15 µl mQ water, and dsRNA concentration was determined spectrophotometrically. The data were normalized so that 100% was assigned to the highest dsRNA amount. The data are present as mean±S.D. of three independent measurements.

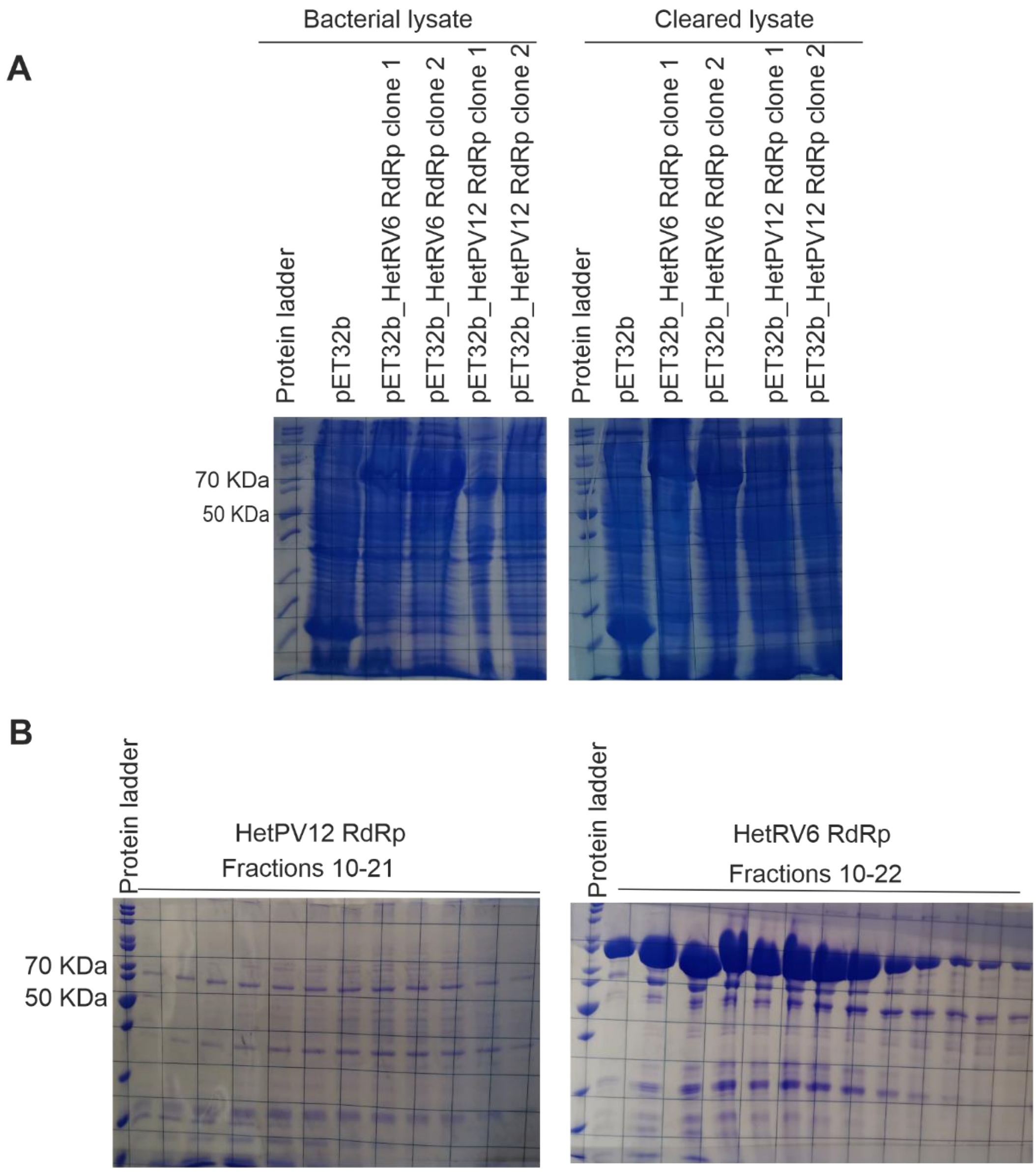

Figure S2. Purification of RdRp of curvulavirus HetRV6 and partitivirus HetPV12. (A) The viral RdRps were cloned into the expression vector pET-32b(+) and the proteins with N-terminal His-tag were expressed in *E. coli* cells. An empty vector was included as a control, and two bacterial clones for each insert were verified for protein expression. All four clones expressed the target proteins (left gel). However, only HetRV6 RdRp was expressed as a soluble protein (right gel). (B) The cleared lysates were applied onto HisTrap HP 1 ml column (GE Healthcare), and peak fractions (or corresponding fractions originating from the HetPV12 RdRp expression) were analyzed in 16% SDS-PAGE gel.

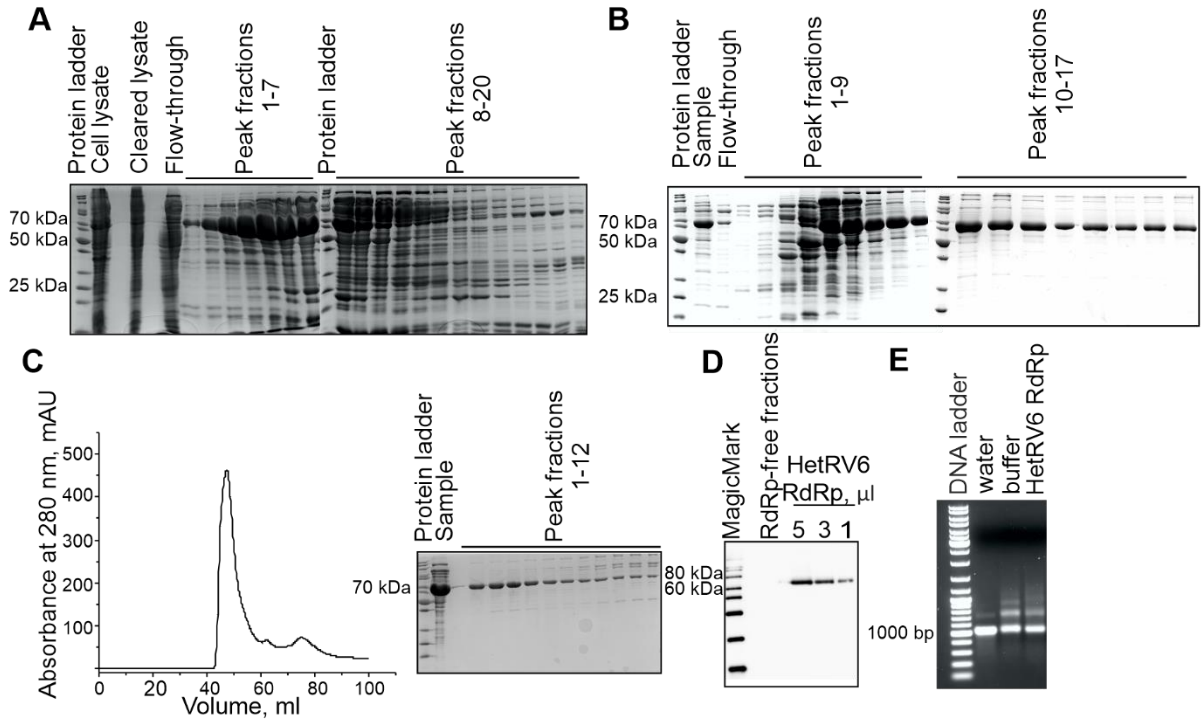

Figure S3. Purification of recombinant HetRV6 RdRp. (A) Non-tagged HetRV6 RdRp was expressed in *E. coli* cells, which were subsequently lysed, and the cleared lysate was loaded onto HiTrap Heparin HP affinity column. A linear 0 to 1M NaCl gradient in 35 column volumes (CV) was applied at 1 ml/min, 1 ml peak fractions were collected, and aliquots of fractions were analyzed by SDS-PAGE. (B) The fractions 1–13 were combined, diluted 10-fold with cold mQ water, and loaded onto HiTrap Q HP anion exchange column. A linear gradient from 0 to 1 M NaCl in 35 CV at 1 ml/min was used to elute proteins and aliquots of collected fractions were analyzed by SDS-PAGE. (C) The fractions 5–17 were combined, loaded onto HiLoad 16/600 Superdex 200 pg gel filtration column, and the HetRV6 RdRp was eluted under isocratic conditions at 1.5 ml/min. The absorbance profile (280 nm) of the eluate (left) and SDS-PAGE analysis of the collected fractions (right) are presented. (D) The fractions 2–6 were combined, concentrated, and the indicated amounts of this preparation were analyzed by Western Blot using RdRp specific antibodies. (E) To confirm that the purified HetRV6 RdRp is not contaminated with RNAases, the RdRp was incubated with HetRV6 RNA1^{+} at 37°C for 1 h. Incubation of the RNA with nuclease-free water and with the reaction buffer was performed as controls for RNA integrity.

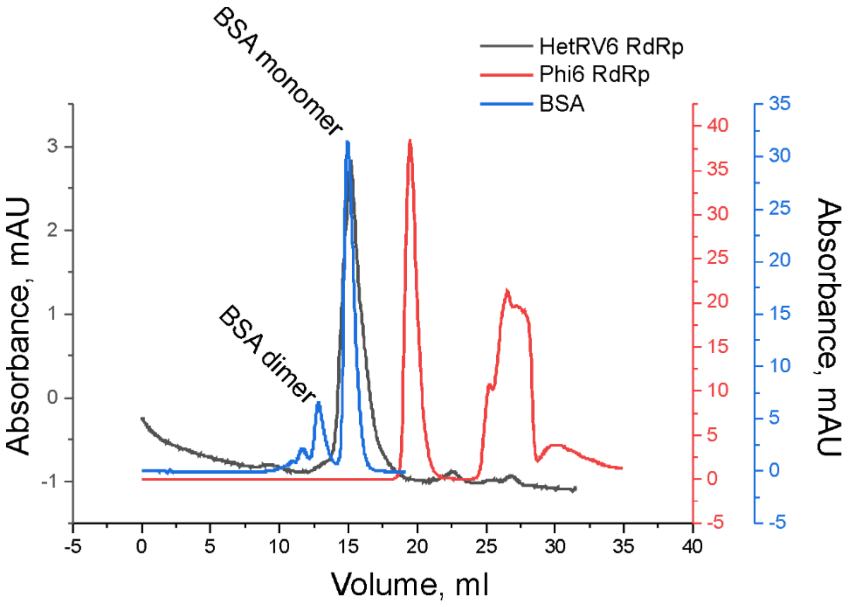

Figure S4. Elution of HetRV6 RdRp, phi6 RdRp, and BSA from gel filtration column. BSA, HetRV6 RdRp or phi6 RdRp were applied onto Superdex 200 10/300GL column (GE Healthcare) followed by isocratic elution with the buffer containing 50 mM phosphate buffer pH8.0, 150 mM NaCl at 0.3 ml/min.

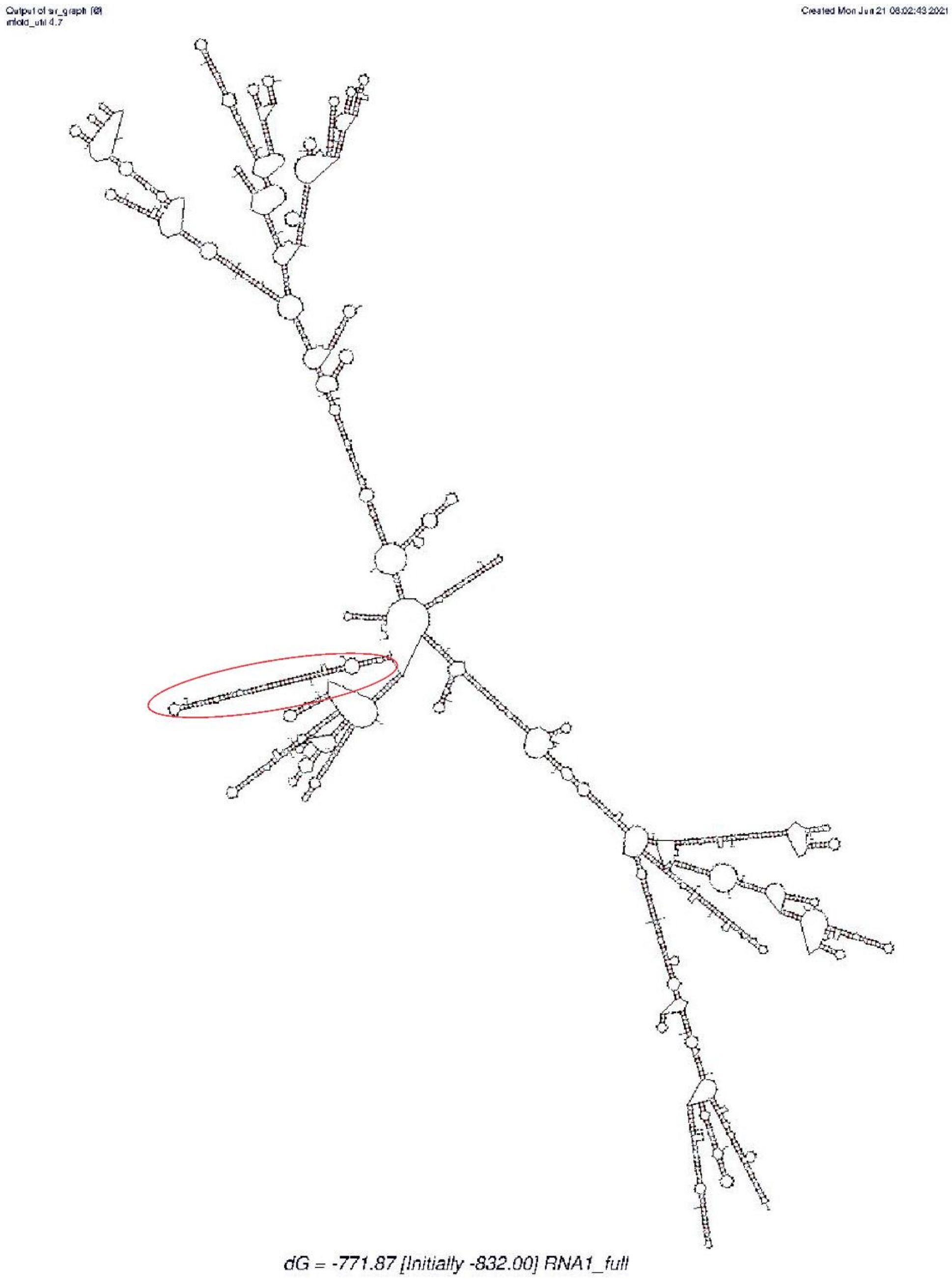

Figure S5. Secondary structure prediction of HetRV6 RNA1+. The 2050 nucleotides long sequence was analyzed with the UNAFold web server (http://www.unafold.org/RNA_form.php) to generate the putative secondary structure. Hairpin at the 3′-end is marked with a red eclipse.

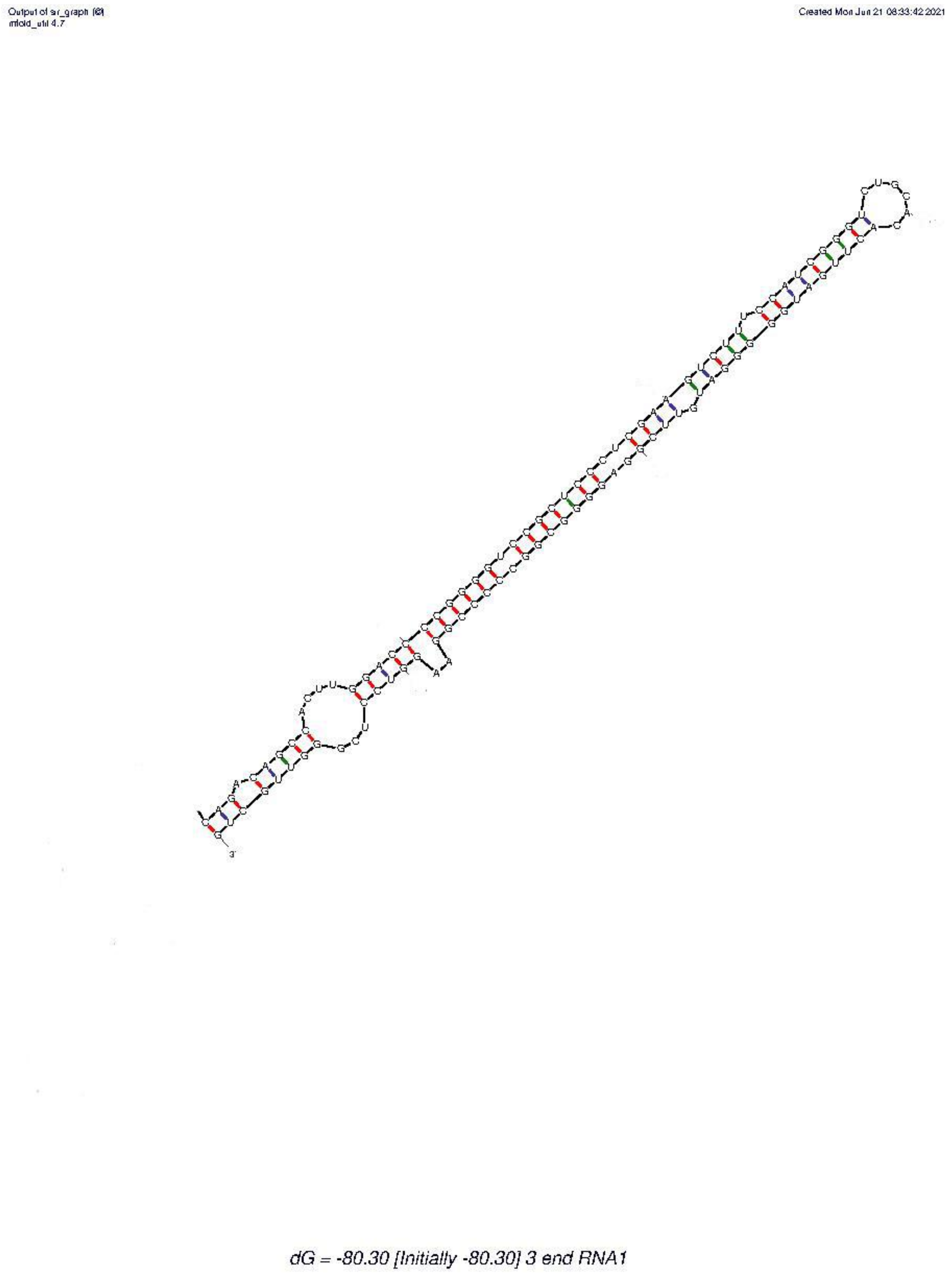

Figure S6. Secondary structure of the 3′-end from HetRV6 RNA1+. A total of 112 nt out of 154 nt from the 3′ untranslated region participate in the formation of the predicted hairpin structure. The structure was generated with the UNAFold web server (http://www.unafold.org/RNA_form.php).

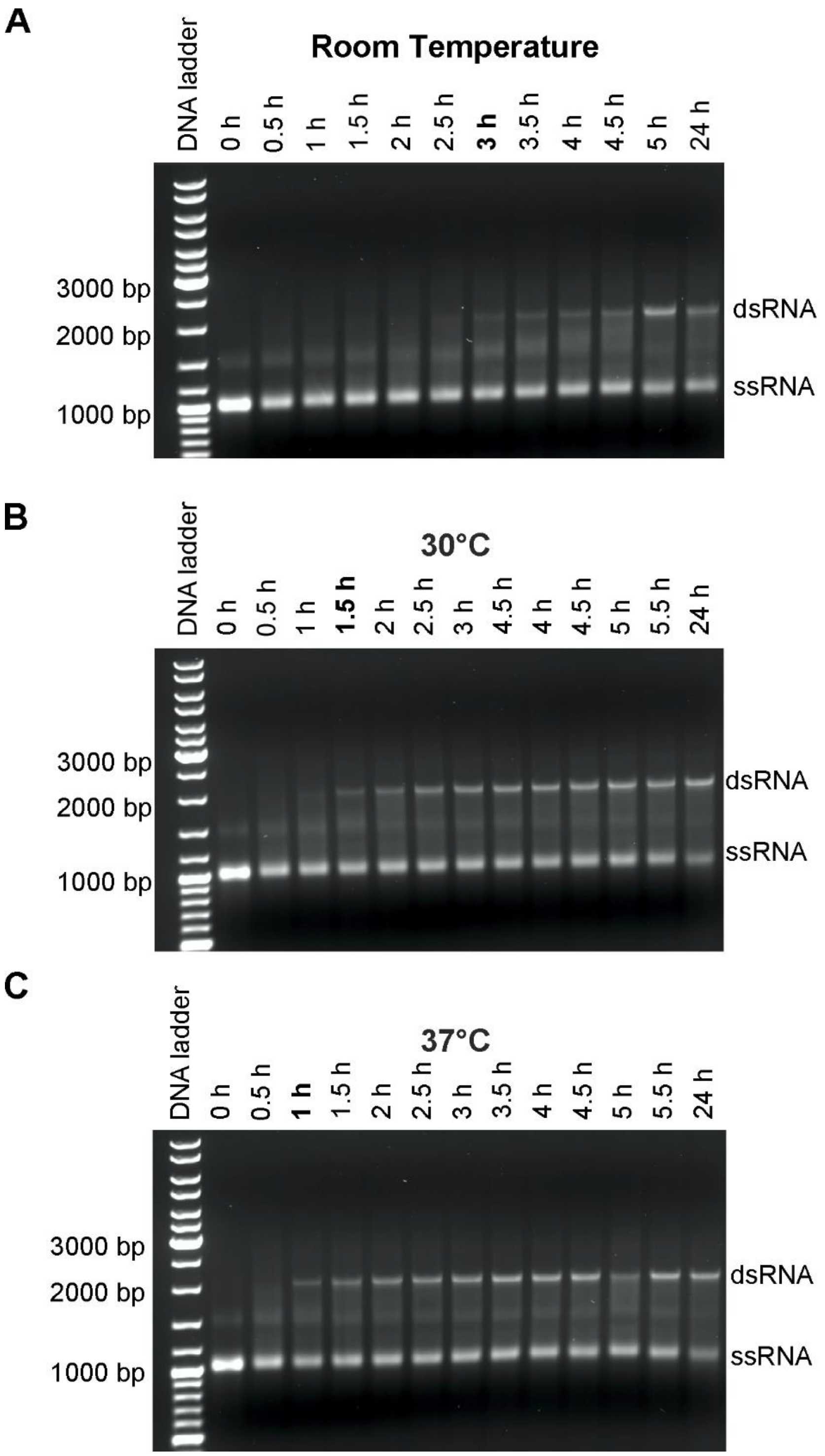

Figure S7. Influence of temperature on the HetRV6 RdRp catalyzed RNA replication rate. Polymerization assay with HetRV6 $RNA1^{+}$ as a template was set up at room temperature (A), 30°C (B), and 37°C (C). In all reactions, 1 mM of each NTP was used. Aliquots of 5 µl were removed from each reaction at the indicated time points and mixed with the equal volume of 2×U buffer to terminate the reaction. The reaction products were analyzed on 1% native agarose gel. The mobility of ssRNA and dsRNA is indicated on the left. The time point at which full-length dsRNA product is clearly visible is indicated in bold.

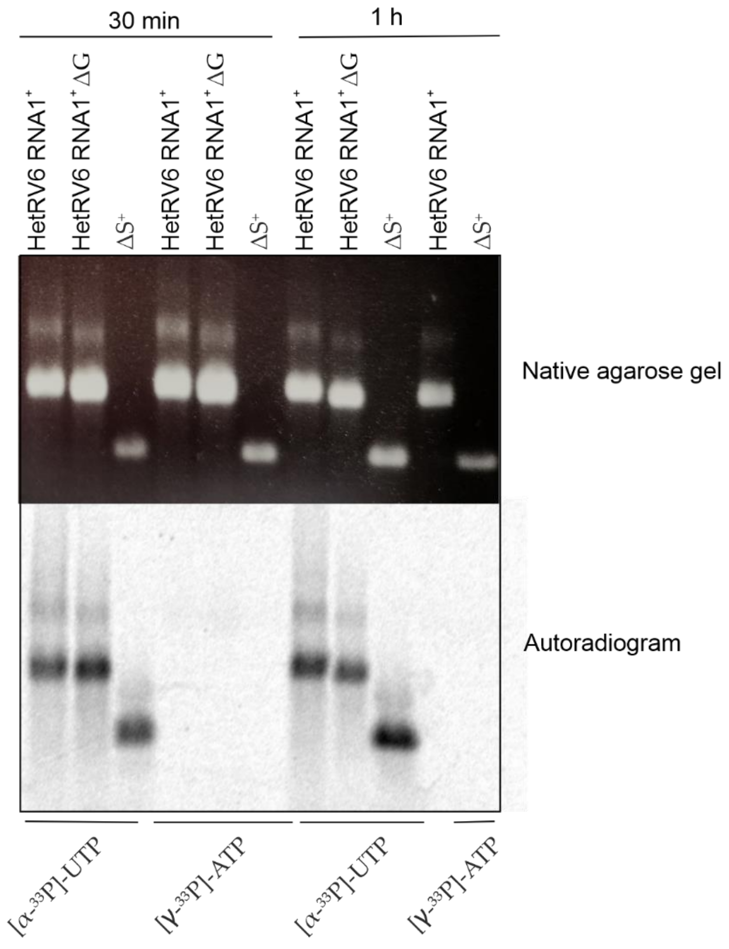

Figure S8. TNTase activity of HetRV6 RdRp. Native HetRV6 RNA1+, its truncated version without G3′, and phi6 Δs+ ssRNA were used as substrates in the TNTase assays catalyzed by HetRV6 RdRp. Autoradiogram of the reactions shows label incorporation from [α-^{33}P]-UTP or [γ-^{33}P]-ATP.

Table S1. Oligonucleotides used in the study

Name	Sequence[1,2]	Description	Reference
Fwd _HRV6_NcoI	GCA**CCATGG**CTTCAACTCCATCCTCATTTCAG	Cloning primer, NcoI site	this study
Rev_HRV6_HindIII	GCA**AAGCTT**CTATCCTCGCCGCTCGGATCAAT	Cloning primer, HindIII site	this study
Fwd_HRV3_NcoI	GCA**CCATGG** CTCAAACCTTACTCTCTGCTGTC	Cloning primer, NcoI site	this study
Rev_HRV3_HindIII	GCA**AAGCTT** CACGCGAAGTCGAGGAAGTGCTT	Cloning primer, HindIII site	this study
HRV6_T7_Fwd_full	TAATACGACTCACTATAGGGCAATAAAGAAGG GACTCAGG	Preparation of cDNA template for HetRV6 RNA1$^+$ production	this study
HRV6_Rev_full	CAGCAACCCGAGGACCTTC	Preparation of cDNA template for HetRV6 RNA1$^+$ production	this study
HRV6_Rev_deltaG	AGCAACCCGAGGACCTCC	Preparation of cDNA template for HetRV6 RNA1$^+$ ΔG production	this study
HRV6_Rev_C	AGCAACCCGAGGACCTCC	Preparation of cDNA template for HetRV6 RNA1$^+$ G->C production	this study
HRV6_T7_Fwd_CDS	TATAGACTCATAGGGATGTCAA CTCCATCCTCATTTCAGA	Preparation of cDNA template for HetRV6 RNA1$^+$ ΔUTR production	this study
HRV6_Rev_CDS	CTATCCTCGCCGCTCGGATCAAT	Preparation of cDNA template for HetRV6 RNA1$^+$ ΔUTR production	this study
3′ end	AGAGAGAGAGCCCCCGA	Primer for preparation of cDNA templates for phi6 s$^+$, Δs$^+$ RNA, and all luc ssRNAs production	Yang et al., 2001
T7-1	CGCGTAATACGACTCACTATAG	Preparation of cDNA template for phi6 s$^+$ and Δs$^+$ RNA production	Yang et al., 2001
3′end_6	GGGAGAGAGAGAGCCCCCGA	Preparation of cDNA template for phi6 Δs$^+$ccc RNA production	Wright et al., 2012
PBV2_T7_Fwd	CGCGTAATACGACTCACTATAG TAAAATTTTCGAATTTTATAATAATTAAG	Preparation of cDNA template for PBV RNA2$^+$ production	Collier et al., 2016
PBV2_Rev	GCAGTTGGGACTGTTAGTCCCAATG	Preparation of cDNA template for PBV RNA2$^+$ production	Collier et al., 2016
pT7_3′end	TAAGCTTGGGCTGCAGGT	Preparation of cDNA template for luc$^+$ RNA	Yang et al., 2001
pT7_3′end_2	CTAAGCTTGGGCTGCAGGT	Preparation of cDNA template for luc$^+$ G RNA	Yang et al., 2001
pT7_3′end_3	GTAAGCTTGGGCTGCAGGT	Preparation of cDNA template for luc$^+$ C RNA	Yang et al., 2001
pT7_3′end_4	TTAAGCTTGGGCTGCAGGT	Preparation of cDNA template for luc$^+$ A RNA	Yang et al., 2001

[1] Restriction enzyme cut sites are in bold

[2] T7 polymerase promoter sequence is underlined

Table S2. Percentages of different nucleotides in the templates ssRNAs

ssRNA template	Nucleotide, %			
	A	C	G	U
HetRV6 RNA1^{+}	22% (451 nt)	26% (525 nt)	31% (639 nt)	21% (435 nt)
HetRV6 RNA1^{+}ΔUTR	22% (402 nt)	25% (461 nt)	32% (575 nt)	21% (383 nt)
PBV2^{+}	28% (481 nt)	20% (352 nt)	26% (457 nt)	26% (455 nt)
phi6 s^{+}	21.5% (633 nt)	27.5% (811 nt)	27.6% (815 nt)	23.4% (689 nt)
phi6 Δs^{+} (phi6 S-segment Δ593—2830)	22% (156 nt)	28% (198 nt)	26% (187 nt)	24%(169 nt)
luc+	29% (532 nt)	21% (383 nt)	23% (426 nt)	27% (483 nt)
